# ECG Abnormalities Preceding Structural Changes of Apical Hypertrophic Cardiomyopathy by up to 10 Years

**DOI:** 10.7759/cureus.42255

**Published:** 2023-07-21

**Authors:** Taylor McManus, John J Straub, Austin Triana, Jose F Triana

**Affiliations:** 1 Cardiology, University of Texas Medical Branch at Galveston, Galveston, USA; 2 Internal Medicine, Vanderbilt University Medical Center, Nashville, USA; 3 Cardiology, Methodist Health System, San Antonio, USA

**Keywords:** diagnosis, pacemaker, ecg, cardiomyopathy, apical hypertrophic cardiomyopathy

## Abstract

A 48-year-old man with hypertension and hypercholesterolemia was referred with an abnormal ECG showing signs of myocardial ischemia and structural change consistent with ventricular hypertrophy. Upon further workup with MRI, echocardiogram, and exercise stress test with perfusion images, it was determined that the man had no cardiac abnormalities. Ten years later, the patient developed structural changes consistent with the abnormal ECG. The patient was diagnosed with apical hypertrophic cardiomyopathy and was treated appropriately with an automatic implantable cardiac defibrillator. The objective of this clinical case report is to highlight this unusual incident where ECG changes preceded structural changes within the heart by 10 years.

## Introduction

Hypertrophic cardiomyopathy (HCM) is an autosomal dominant cardiovascular disorder primarily affecting the left ventricular wall. This condition is estimated to affect approximately two out of every 1000 people [1]. To be diagnosed with hypertrophic cardiomyopathy, the left ventricle must have a thickness of over 15 mm in the absence of any other cause of left ventricular hypertrophy (LVH) [2]. Apical hypertrophic cardiomyopathy (AHCM or Yamaguchi Syndrome) is a variant of hypertrophic cardiomyopathy with a similar level of interstitial fibrosis and less myocyte disorganization. The clinical criteria for AHCM include asymmetric LVH predominantly at the apex (>15 mm) along with a > 1.5 ratio of apical to basal wall thickness [3]. The fact that this condition presents similarly to other variants of HCM and acute coronary disease makes it difficult for clinicians to consistently diagnose, although presentation and diagnostics do differ slightly. The median presenting age for classic HCM is 46 while the apical variant has a slightly earlier presenting age at 41. AHCM is inherited in an autosomal dominant pattern; however, studies have shown that the condition has variable expressivity as well as age-dependent variations in penetrance [4]. Symptoms of AHCM generally include dyspnea, atrial fibrillation (reported in 20% of patients), chest pain, exercise intolerance, and syncope/presyncope [1]. Evaluation of ECG in these two subsets demonstrates different wave patterns, with giant negative T-waves (> or = 1 mV) characteristic of AHCM, whereas classic HCM has nonspecific ST-segment and T-wave anomalies. Both conditions present with large QRS complexes in leads V2-V6, which is indicative of LVH [5]. As rare as HCM is, the apical variation is exceedingly rare and presents mainly in Japanese individuals (15% of AHCM individuals as opposed to only 3% presenting in Caucasians) [6]. In the existing literature, structural and ECG findings are typically present concurrently.

## Case presentation

**Figure 1:**
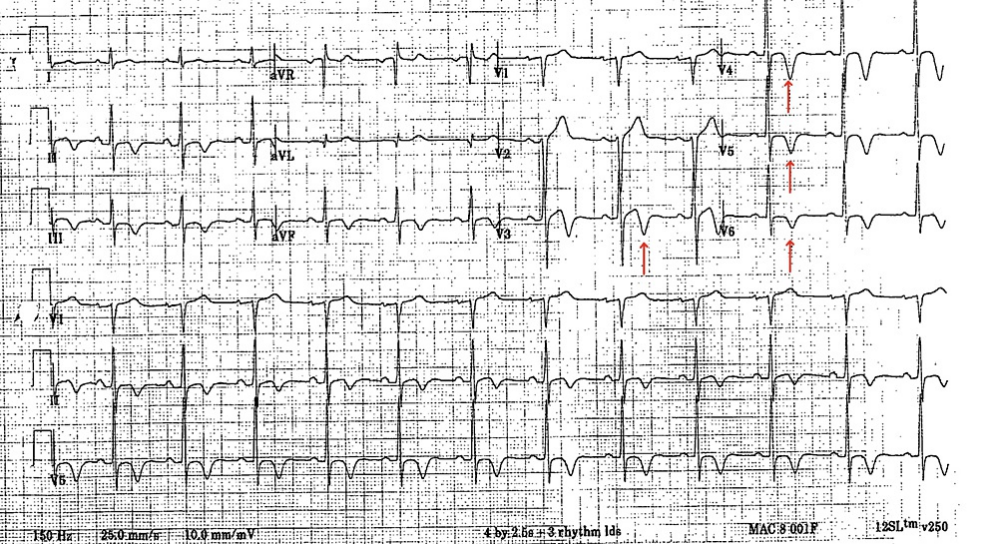
2003 electrocardiogram (ECG) showing first indication of abnormalities

A 48-year-old man with a history of hypertension and hypercholesterolemia presented for evaluation of an abnormal ECG (Figure 1), which showed T-wave inversion in the inferolateral leads with suspicion of myocardial ischemia and not present on prior ECGs of years earlier. He denied symptoms, including chest pain, palpitations, syncope, cough, or hemoptysis. The patient has a family history of premature coronary artery disease, but no evidence of hereditary structural cardiovascular conditions. The patient’s blood work was within normal limits, and chronic conditions were stable. No structural abnormalities were identified through cardiac MRI, echocardiogram, and exercise and nuclear stress testing. Initial cardiac MRI showed an end-diastolic interventricular septal wall thickness of 13.4 mm. The MRI did not show evidence of infiltrative disease. The patient did not develop any symptoms over the next decade but remained under close follow-up with adequate control of his hypertension with hydrochlorothiazide and lisinopril, as well as dietary changes.

**Figure 2:**
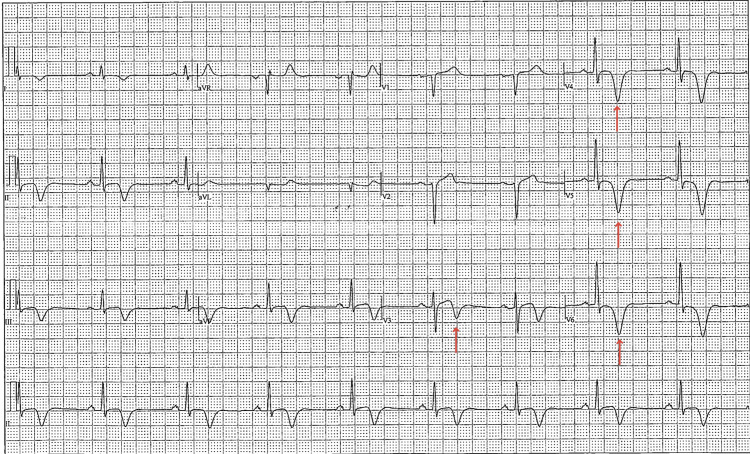
2021 electrocardiogram (ECG) after implantable cardioverter defibrillator (ICD) implantation showing worsening negative T waves in inferolateral leads, 18 years after initially abnormal ECG

Ten years after the initial echocardiogram, the patient developed new structural abnormalities, including thickening of the apical membrane corresponding to the abnormal ECG, which had also worsened (Figure 2). Cardiac MRI (Video 1) measured apical anterior septal wall thickness at 12.5 mm and inferior wall at 9.6 mm during end diastole (previously 9.6 mm and 9.2 mm, respectively). His interventricular septal wall thickness also progressed to 12.3 mm with late gadolinium enhancement in the LV apex, and his apical region at the papillary muscle insertion point showed measurements of 15.8 mm. LVEF was measured at approximately 55%. A transthoracic echocardiogram (TTE) performed at the time of diagnosis observed demonstrated abnormal apical hypertrophy, Throughout this discovery, the patient remained asymptomatic.

**Video 1:** 2013 cardiac MRI demonstrating abnormalities The video content is played twice for ease of viewing. This MRI was the first structural indication of abnormal findings.

The patient was subsequently diagnosed with AHCM and was treated with an implantable cardioverter-defibrillator and atenolol due to the presence of paroxysmal ventricular tachycardia and increased risk of sudden cardiac death. This case describes a situation where the ECG changes preceded the structural abnormalities by 10 years. A similar patient has not been described to our knowledge in any existing literature.

## Discussion

The apical variant of HCM is seen less frequently in the population compared to classical hypertrophic cardiomyopathy so data regarding this specific condition is somewhat limited. In addition, it is currently undescribed in literature to see ECG change prior to the development of any structural abnormalities within the heart. Our patient, who demonstrated a focal area of myocardial thickening predominantly in an apical distribution, has a particularly noteworthy case due to the onset of ECG abnormalities up to 10 years prior to the development of structural abnormalities. Currently, the diagnostic criteria include both ECG changes and concurrent left ventricular hypertrophy predominantly in the apex [7]. Such a case where there is a significant amount of time between ECG changes and the development of structural abnormalities may suggest a need to reconsider diagnostic criteria. One study detailed a similar case where abnormal ECG changes with an asymptomatic patient led to a diagnosis of hypertrophic cardiomyopathy; however, this patient experienced mild left ventricular hypertrophy, a reduced ejection fraction, left ventricular dilation, and mild LV ischemia [7]. The comparison between our patient and such cases lies in the complete absence of structural changes consistent with AHCM for 10 years.

We believe this case supports the hypothesis that the ECG changes are not due to relative ischemia related to ventricular hypertrophy [8]. Rather, there is possibly a separate Purkinje-myocardium coupling abnormality leading to patchy activation of the myocardium or mechanistic changes to cardiac ion channels. It is unclear whether there is a genetic component or if there was an unidentified, environmental insult that precipitated these changes. Clinically, this patient may be at higher risk for arrhythmia and sudden cardiac death. Recent literature suggests that deep T-wave inversions in apical HCM, as they appear similarly to acute coronary syndrome, tip off physicians for further workup. Additionally, up to 50% of these individuals are diagnosed solely off ECG findings that are noted incidentally [9].

## Conclusions

The development of structural changes after nearly a decade of abnormal ECG findings without physical cardiac abnormality is the first reported case of its kind. The patient’s condition was discovered swiftly as a result of regular echocardiogram screenings for years prior to its development. Future research into ECG changes preceding cardiovascular conditions may also be warranted to advance our understanding of cardiovascular health and diagnostic tools like the ECG. Investigating whether other cardiac conditions experience diagnostic ECG changes before the development of a disorder could also be beneficial to the specialty.
